# Four-compartment muscle fatigue model to predict metabolic inhibition and long-lasting nonmetabolic components

**DOI:** 10.3389/fphys.2024.1366172

**Published:** 2024-03-11

**Authors:** Florian Michaud, Santiago Beron, Urbano Lugrís, Javier Cuadrado

**Affiliations:** Laboratory of Mechanical Engineering, Centro de Investigación en Tecnologías Navales e Industriales (CITENI), Campus Industrial de Ferrol, Universidade da Coruña, Ferrol, Spain

**Keywords:** musculotendon dynamics, muscle force, muscle fatigue model, force prediction, musculotendon model, sport performance, ergonomics, mathematical models

## Abstract

**Introduction:**

Computational muscle force models aim to mathematically represent the mechanics of movement and the factors influencing force generation. These tools allow the prediction of the nonlinear and task-related muscle behavior, aiding biomechanics, sports science, and rehabilitation. Despite often overlooking muscle fatigue in low-force scenarios, these simulations are crucial for high-intensity activities where fatigue and force loss play a significant role. Applications include functional electrical stimulation, motor control, and ergonomic considerations in diverse contexts, encompassing rehabilitation and the prevention of injuries in sports and workplaces.

**Methods:**

In this work, the authors enhance the pre-existing 3CCr muscle fatigue model by introducing an additional component of force decay associated with central fatigue and a long-term fatigue state. The innovative four-compartment model distinguishes between the short-term fatigued state (related to metabolic inhibition) and the long-term fatigued state (emulating central fatigue and potential microtraumas).

**Results:**

Its validation process involved experimental measurements during both short- and long-duration exercises, shedding light on the limitations of the traditional 3CCr in addressing dynamic force profiles.

## 1 Introduction

Inspired by the first Hill’s biomechanical theories, computer modeling and simulation of muscle forces aim to mathematically represent the complex interactions within muscles during contraction (Herzog et al., 2015). They serve as valuable tools for understanding and predicting muscle behavior, providing insights into the mechanics of movement and the factors influencing force generation. By simulating various physiological conditions and scenarios, these models aid researchers in unraveling the intricacies of muscle function, facilitating advancements in biomechanics, sports science, and rehabilitation (Mathieu et al., 2023; Michaud et al., 2021; Lamas et al., 2022). Historically, simulation of muscle forces has often overlooked the influence of muscle fatigue, particularly in scenarios characterized by low-force requirements. While such oversights may be inconsequential for some tasks, they become crucial in high-intensity activities where muscle fatigue and subsequent force loss are expected. These simulations offer valuable applications in areas such as functional electrical stimulation (FES) (Levy et al., 1993), motor control and prediction, and addressing ergonomic considerations where the dynamic estimation of muscle force over time is crucial. This requirement is evident in diverse contexts, encompassing rehabilitation, the prevention of injuries in sports and workplaces, and the strategic planning for the surgical reconstruction of diseased joints.

Muscle fatigue cannot be modeled as a single universal mechanism, since it follows nonlinear behavior, is task-related, and can vary across muscles and joints (Enoka and Duchateau, 2008). Various fatigue modeling approaches have been presented in the literature (Ding et al., 2000; Ding et al., 2003; Xia and Frey Law, 2008; Ma et al., 2009; Pereira et al., 2011; Giat et al., 1996). One of them, the three-compartment controller (3CC) model (Frey-Law et al., 2012) marked an improvement over an earlier model that could only represent maximum activation (Liu et al., 2002). Xia and Frey-Law introduced this model equipped with a feedback controller to match target loads within a single muscle or at joint level, thus allowing it to handle any time-varying force profile (Looft et al., 2018). Consequently, this fatigue model offers a relatively straightforward and adaptable solution for various applications.

Over the past decade, there has been a growing interest in integrating muscle fatigue into predictive models for task accomplishment which reflects a significant emphasis on injury prevention in both sports and workplace settings. As a result, several studies have utilized the flexibility of the 3CC model by integrating it into their simulations of muscle force at the joint level (Barman et al., 2022; Sonne and Potvin, 2016), or by accounting for redundant muscle forces within a multibody environment (Michaud et al., 2023; Mohamed Refai et al., 2024; Pereira et al., 2011). While the 3CC approach has been evaluated and validated (Liu et al., 2002; Looft et al., 2018; Lugrís et al., 2023), the authors of this paper identified limitations when applying this model to predict muscles forces for short-duration high-intensity exercise (Michaud et al., 2023). Despite subject specific calibration from a short-term exercise protocol, the simulation failed to accurately capture the expected force decay during a long-term training session. The model reached a stable equilibrium, suggesting that a certain level of activity could be sustained indefinitely. This asymptote has been linked in a previous publication to fatigue and recovery ratios, particularly for sustained static tasks (Frey-Law et al., 2012).

It must be noted that the 3CC was originally designed to model peripheral muscle fatigue and recovery, focusing primarily on metabolic factors. Therefore, a quick recovery could be expected. This recovery during the resting period was later modified and enhanced by the same and other investigators by means of the 3CCr model in (Looft et al., 2018), by incorporating a recovery factor to better match published experimental results. Nevertheless, both Baker et al. and Jubeau et al. (Baker et al., 1993; Jubeau et al., 2012), highlighted that, when observing a high number of repetitions, nonmetabolic factors and long-term fatigue are also involved in muscle fatigue. By calibrating the 3CC parameters, as done in (Frey-Law et al., 2021), to match the fatigue history observed in Jubeau et al. (Jubeau et al., 2012) during the repetitions, we found that the muscle fatigue was fully recovered (99.8%) after 1 h of rest. Experimental results, however, showed that fatigue recovery was around 75% after 1 h and slightly over 80% after 24 h. This observation confirmed the hypothesis made by the authors of this paper in (Michaud et al., 2023), where they suggested a modification of the three-compartment controller model to add a long-term fatigue state.

For this reason, the purpose of this study is to enhance the existing 3CCr muscle fatigue model by integrating an additional force decay component that corresponds to central fatigue (often referred to as “brain effort”) and a “long-term fatigue state” (fatigue from which the subject recovers only after a long resting period). Specifically, the authors introduced a four-compartment model that distinguishes between the short-term fatigued state (corresponding to metabolic inhibition) and the long-term fatigued state (simulating central fatigue and potential microtraumas). Through new experimental measurements during short and long-duration exercises, they introduced a new methodology to estimate the subject-specific fatigue parameters of their model. They validated their approach, and also demonstrated the limitations of the classic 3CCr in matching target loads within a single muscle or at the joint level, highlighting its inability to handle any time-varying force profile.

## 2 Material and methods

### 2.1 Experimental data collection

#### 2.1.1 Participants

Seven subjects (4 males, 3 females, age 31 ± 5, height 175 ± 10 cm, body mass 65 ± 15 kg) were recruited for this study involving two sessions. None of the participants were involved in intensive training, while all of them were healthy, without any upper limb injury history or neuromuscular diseases. They provided written informed consent as approved by the Research Ethics Committee of La Coruña-Ferrol prior to all participation.

#### 2.1.2 Instrumentation

**FIGURE 1:**
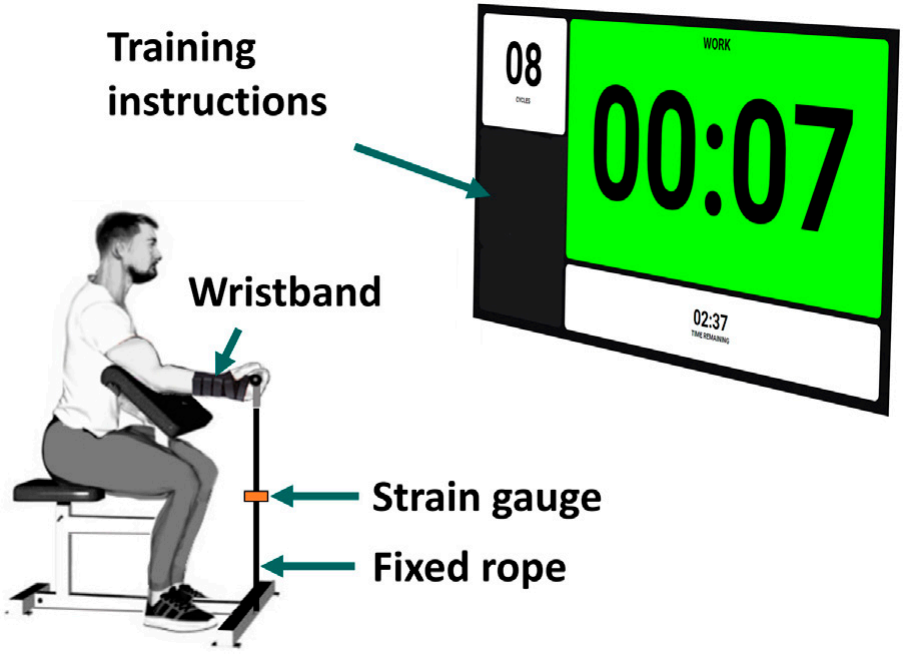
Experimental instrumentation.

Each subject was wearing a wristband to immobilize the wrist and was seated on a preacher arm curl bench, with the upper arm secured at a 45° shoulder flexion to the armrest to minimize force generation from other joints. The elbow was positioned at a 45° flexion, and the wrist was in a supinated position (Figure 1). Participants were asked to perform maximal voluntary isometric contractions (MVCs) of elbow flexion with their dominant arm, assessed by pulling on a fixed rope in series with a strain gauge (Phidgets Micro Load Cell 0–50 kg, sampled at 100 Hz). The measured forces were filtered by singular spectrum analysis (SSA) with a window length of 60 ms.

#### 2.1.3 Experimental procedures

The procedure closely followed that outlined by Baker et al. in (Baker et al., 1993) to identify both metabolic inhibition and long-lasting nonmetabolic component effects. Participants were instructed to perform a short-duration exercise protocol at session #1 and a long-duration exercise protocol at session #2, with a minimum separation of 1 week between the two sessions. At the beginning of each session, subjects completed a 5-min warm-up using a resistance band and carried out a series of elbow flexions at a submaximal level to minimize the risk of injury. Additionally, to become familiar with the isometric evaluation and the instructions, a simulated recording was performed. Instructions (number of repetitions, rate, rest periods, etc.) for each task were provided verbally and visually, displayed on a big screen situated in front of the subject (Figure 1).

**FIGURE 2:**
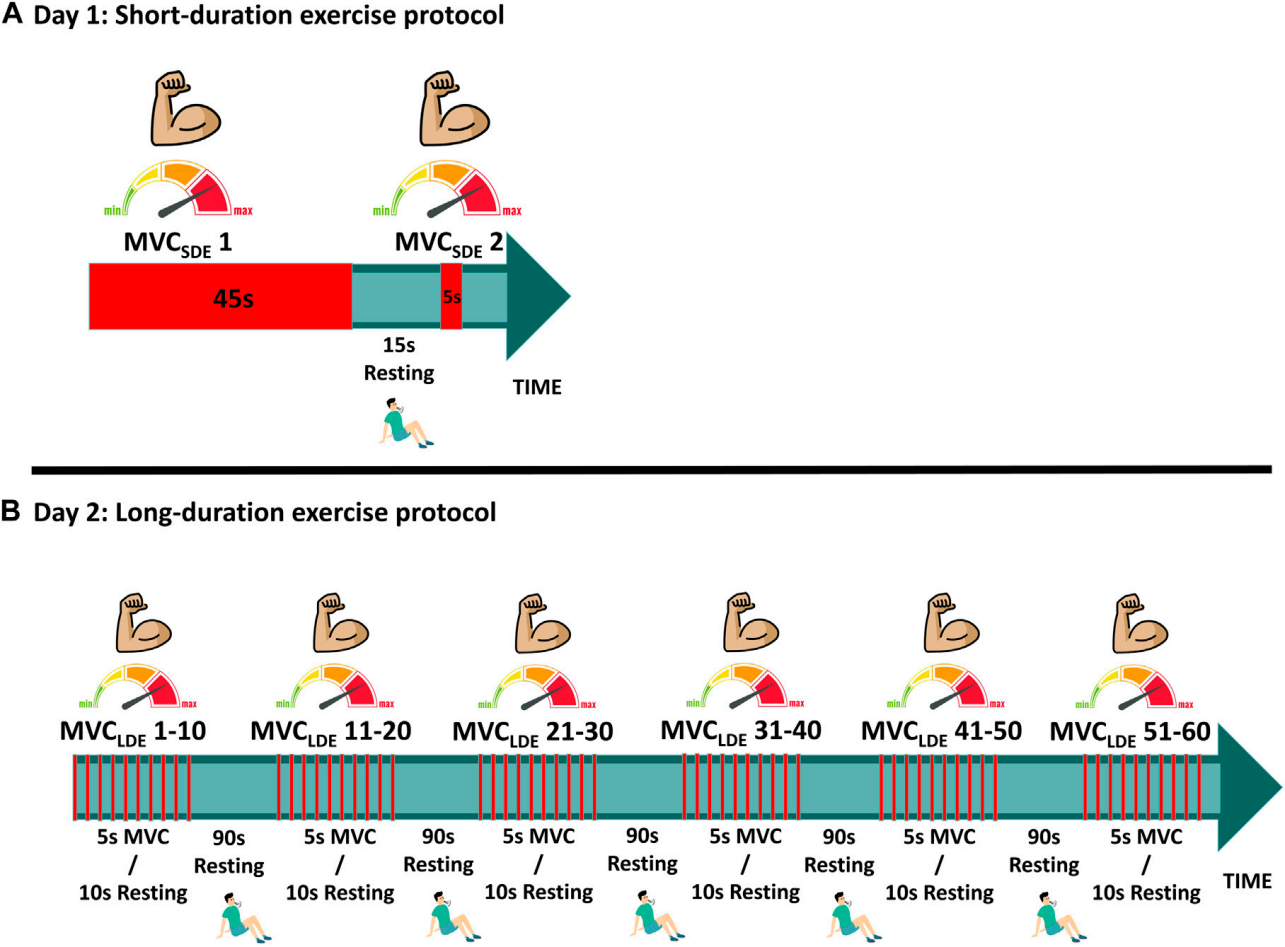
Experimental protocols: **(A)** Short-duration exercise (SDE); **(B)** Long-duration exercise (LDE).

The short-duration exercise protocol (SDE) was performed on session #1, involving two isometric maximum voluntary contractions assessed at baseline (MVC_SDE_) to be used for model parameter identification of metabolic inhibition (Figure 2). The first contraction lasted 45 s (MVC_SDE_1), followed by a 15-s rest; the second contraction was sustained for 5 s (MVC_SDE_2). To assess the fatigue recovery process, an additional MVC, sustained for 5 s, was performed after a resting period of 10 min (MVC-R1) following the conclusion of MVC_SDE_2.

The long-duration exercise protocol (LDE) was performed on session #2, with some deviations from the procedures outlined by Jubeau et al. (Jubeau et al., 2012) and Xenofondos et al. (Xenofondos et al., 2018). The protocol consisted of 60 isometric maximum voluntary contractions (MVC_LDE_), each lasting for 5 s, with a 10-s resting period between contractions (Figure 2). However, additional resting periods of 90 s were introduced after the 10th, 20th, 30th, 40th, and 50th repetitions to reduce potential muscle soreness, highlight the effect of the resting period on recovery, and enable the observation of potential muscle potentiation compared to the protocol conducted in (Jubeau et al., 2012). To evaluate the fatigue recovery process, an additional MVC, sustained for 5 s, was conducted after resting periods of 10 min, 1 h, and 24 h (MVC-R1, MVC-R2 and MVC-R3, respectively) counted from the end of the 60th MVC_LDE_. MVC-R2 and MVC-R3 were not conducted during SDE because all subjects exhibited recoveries close to 100% after MVC-R1.

Both sessions were simulated using the full muscle fatigue models, including the resting periods and the MVC evaluations.

### 2.2 Four-compartment muscle fatigue model

Muscle fatigue encompasses both physiological and psychological aspects, representing a reduction in maximal force or power production in response to contractile activity. It can manifest at various levels of the motor pathway, typically categorized into central and peripheral components. Central fatigue originates from the central nervous system (CNS), leading to a decline in neural drive to the muscle (Gandevia, 2001). On the other hand, peripheral fatigue occurs at the neuromuscular junction and within the muscle, involving mechanical and cellular changes (WALLMANN, 2007).

To systematically assess muscle peripheral fatigue related to complex or dynamic movements, Xia and Frey-Law proposed the so-called three-compartment model described in (3CC) (Xia and Frey Law, 2008) with subsequent enhancements detailed in (Frey-Law et al., 2012; Frey-Law et al., 2021). This model serves to characterize muscle activation (*M*_*a*_), fatigue (*M*_*f*_), and recovery (*M*_*r*_) across any loading conditions. The cumulative percentage of motor units (MU) in each compartment totals 100%. During activity, motor units from the resting compartment transition to the activated compartment at a rate regulated by a feedback controller, *C(t)*, aligning with the target load (*TL*) expressed as a percentage of the maximum voluntary contraction (%MVC). Additionally, this controller facilitates the reverse movement of motor units (from *M*_*a*_ to *M*_*r*_) if an excess of units is activated beyond the requirement to match a specific *TL*. Later, the same and other investigators modified and enhanced the recovery rate during the rest period under the 3CCr model in (Looft et al., 2018), by incorporating a recovery factor (*r*) to better match published experimental results.

While the effectiveness of the 3CC and 3CCr approaches has been verified and validated (Looft et al., 2018; Sonne and Potvin, 2016), the authors of this paper identified limitations when applying this model to the prediction of muscle forces during short-duration, high-intensity exercises (Michaud et al., 2023). Despite parameter calibration using a short-term exercise protocol, the simulation failed to accurately depict the measured force decay during an extended training session. The model reached a stable equilibrium, implying that a certain level of activity could be sustained indefinitely. Previous publications have linked this asymptote to fatigue and recovery ratios (*F* and *R*), expressed as (1/(*F/R* + 1)) ∗ 100%, especially for sustained static tasks (Frey-Law et al., 2012).

It is essential to recognize that the 3CC was initially designed to model peripheral muscle fatigue and recovery, primarily focusing on metabolic factors. Therefore, a quick recovery is expected. This recovery was later augmented during the rest period in (Frey-Law et al., 2012) by incorporating a recovery factor (3CCr). However, both Baker et al. (Baker et al., 1993; Jubeau et al., 2012) emphasized that, when observing a high number of repetitions, nonmetabolic factors and long-term fatigue also contribute to muscle fatigue. By calibrating the 3CCr parameters, as performed in (Frey-Law et al., 2021), to align with the fatigue history observed in Jubeau et al. (2012) during the repetitions, we determined that muscle fatigue was fully recovered (99.8%) after 1 hour of rest. Nevertheless, experimental results indicated that fatigue recovery was approximately 75% after 1 hour and slightly over 80% after 24 hours. This observation confirmed the hypothesis presented by the authors of this paper in (Michaud et al., 2023), where they proposed a modification to the 3CCr model by introducing a long-term fatigue state.

**FIGURE 3:**
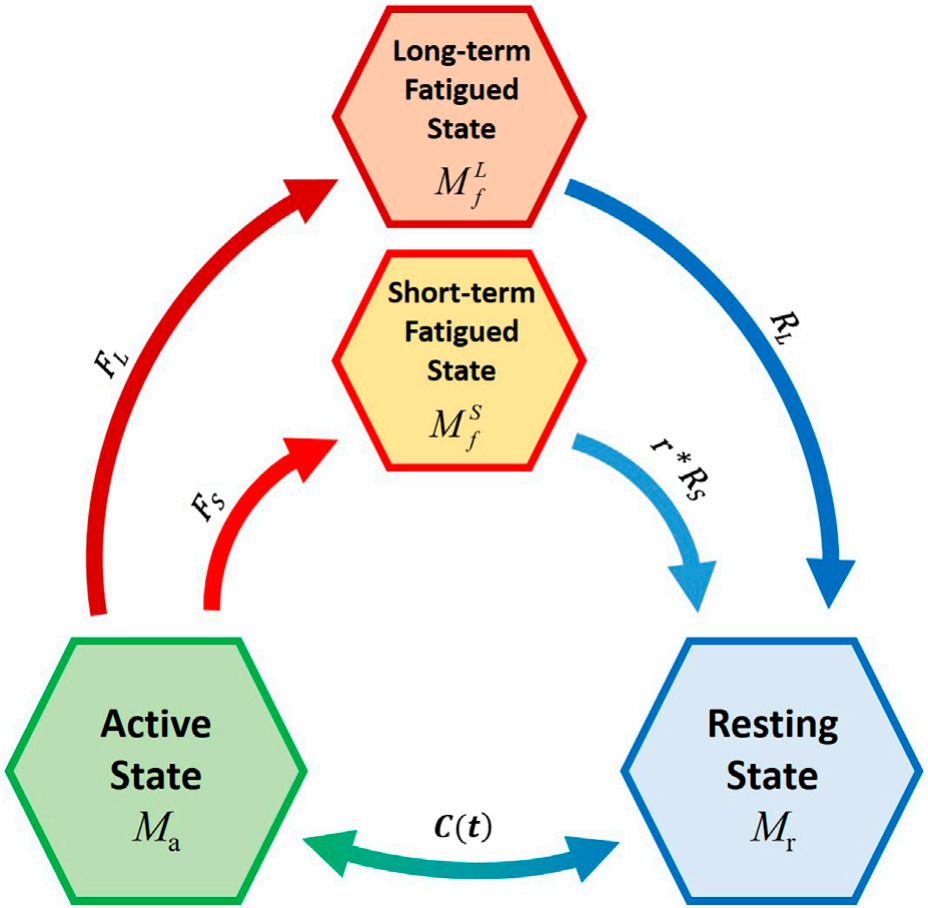
Schematic representation of the novel four-compartment controller (4CC) mathematical fatigue model Adapted with permission from (Frey-Law et al., 2012), where *C*(*t*) is the feedback controller used to match *M*_*a*_ to target load, *TL*; *F*_*S*_ and *F*_*L*_ define the fatigue rate, while *R*_*S*_ and *R*_*L*_ define the recovery rates of the short-term and long-term fatigued states, respectively. *r* is a multiplier to augment recovery during rest (Looft et al., 2018).

Consequently, the new model is composed of four compartments. The muscle active (*M*_*a*_) and recovery (*M*_*r*_) states were maintained from the 3CCr, while the fatigue compartment (*M*_*f*_) was divided into two parts (Figure 3): the short-term fatigued state ( $M_{f}^{S}$ ) and the long-term fatigued state ( $M_{f}^{L}$ ) [see Equations (1)–(5a)]. The sum of percentage of MU in each compartment equals 100% and the flows between the four compartments are mathematically described by differential equations as follows:

$$\frac{dM_{r}}{dt}=-C\left(t\right)+\left(R_{S}\times r\right)\times M_{f}^{S}+R_{L}\times M_{f}^{L}$$ (1)

$$\frac{dM_{a}}{dt}=C\left(t\right)-\left(F_{S}+F_{L}\right)\times M_{a}$$ (2)

$$\frac{dM_{f}^{S}}{dt}=F_{S}\times M_{a}-\left(r\times R_{S}\right)\times M_{f}^{S}$$ (3)

$$\frac{dM_{f}^{L}}{dt}=F_{L}\times M_{a}-R_{L}\times M_{f}^{L}$$ (4)

where,

Ct=TL−Ma when Ma<TL and Mr>TL‐Ma (5a)

Ct=Mr when Ma<TL and Mr<TL‐Ma (5b)

$$C\left(t\right)=\left(TL-M_{a}\right)\text{ when }M_{a}>TL$$ (5c)

r=10 when TL=0; if not,r=1 (5d)

*F*_*S*_ and *F*_*L*_ define the fatigue rate, while *R*_*S*_ and *R*_*L*_ define the recovery rates of the short-term and long-term fatigued states, respectively. *r* is a multiplier to augment recovery during rest (Looft et al., 2018).

As all non-fatigued muscle units can be recruited, the percentage of each subject’s maximum force available at the joint level is:

$$F^{\mathrm{Max}}=100-\left(M_{f}^{S}+M_{f}^{L}\right).$$ (6)

### 2.3 Subject-specific calibration

While there are normative, joint-specific values identified for the 3CC coefficients for average behavior (Xia and Frey Law, 2008; Looft et al., 2018), they are not subject-specific. In this study, the authors proposed a novel methodology to allow the calibration of these coefficients. In this preliminary validation, the authors applied their novel approach at the joint-level for fatigue prediction.

#### 2.3.1 %MVC

To enable comparison among subjects and with other studies, as well as to utilize the 3CC model, a relative unit-less measure of muscle force, expressed as a percentage of maximum voluntary contraction (%MVC), was employed in this work. The maximal voluntary isometric contraction strength during the first MVC of each session served as the reference for the force histories within the same session.

#### 2.3.2 Short-term fatigued states parameters

The calibration of parameters *F*_*S*_ and *R*_*S*_ for the short-term fatigued state was performed using experimental measurements from the SDE protocol. We hypothesized that during this brief session, long-term fatigue effects could be omitted, so the fourth compartment was not taken into account. The fatigue parameters were considered the same for all the muscles of the joint, and were calibrated from recorded activities at joint level by means of optimization (*fmincon*, Matlab) seeking to best fit model and experimental results, similar to what was proposed by Frey-Law et al. in (Frey-Law et al., 2021). *F*_*S*_ and *R*_*S*_ were the design variables of the optimization problem which attempted to minimize the residuals between model estimates of decaying MVC (*M*_*a*_ during MVC trials) and observed MVCs (force measurements). MVC_SDE_1 measurements provided optimal visibility of force decay (for adjusting *F*_*S*_) despite the pronounced noise in force measurements. By sustaining the effort over a prolonged time period, MVC_SDE_1 minimized the discrepancies observed when using shorter durations. MVC_SDE_2 was necessary to calibrate the recovery parameter after an effort (for adjusting *R*_*S*_).

#### 2.3.3 Long-term fatigued states parameters

Conversely, after fixing *F*_*S*_ and *R*_*S*_, the calibration of parameters *F*_*L*_ and *R*_*L*_ for the long-term fatigued state was performed using experimental measurements from the sixty MVC_LDE_ of the LDE protocol, MVC-R1 and MVC-R2. Similarly, *F*_*L*_ and *R*_*L*_ were the design variables in the optimization problem aiming to minimize the residuals between model estimates of decaying MVCs.

### 2.4 4CC validation and 3CC comparison

The results obtained from the classical 3CCr approach served as a benchmark in this study for validating the new model. The precision of both approaches in simulating muscle fatigue during short-duration and long-duration protocols was assessed. However, recognizing the potential significance of subject-specific calibration, the 3CCr parameters underwent calibration through two distinct procedures. In the first approach (3CC-SDE), the authors calibrated the 3CCr muscle fatigue parameters using the identical experimental data employed for calibrating the short-term fatigued states parameters. In the second approach (3CC-LDE), they calibrated the 3CCr muscle fatigue parameters using the same experimental data used for the calibration of the long-term fatigued states parameters.

Estimated maximum muscle forces were compared with strain gauge readings during MVCs, because it is assumed that all muscles are fully activated during a maximum effort, thus avoiding the muscle force sharing problem and the uncertainty on the level of effort made by the subject. The root-mean-square error (RMSE) was calculated between measured and simulated results to quantitatively compare the 4CC, the 3CC-SDE and the 3CC-LDE approaches. The RMSE was evaluated for all the measurements (RMSE Tot.), and also separately for the exercise phase (RMSE Ex.) and the recovery evaluations (RMSE Rec.) at MVC-R1 to MVC-R3 (only MVC-R1 for SDE) during the short-duration and long-duration exercise.

## 3 Results

### 3.1 Experimental readings

**FIGURE 4:**
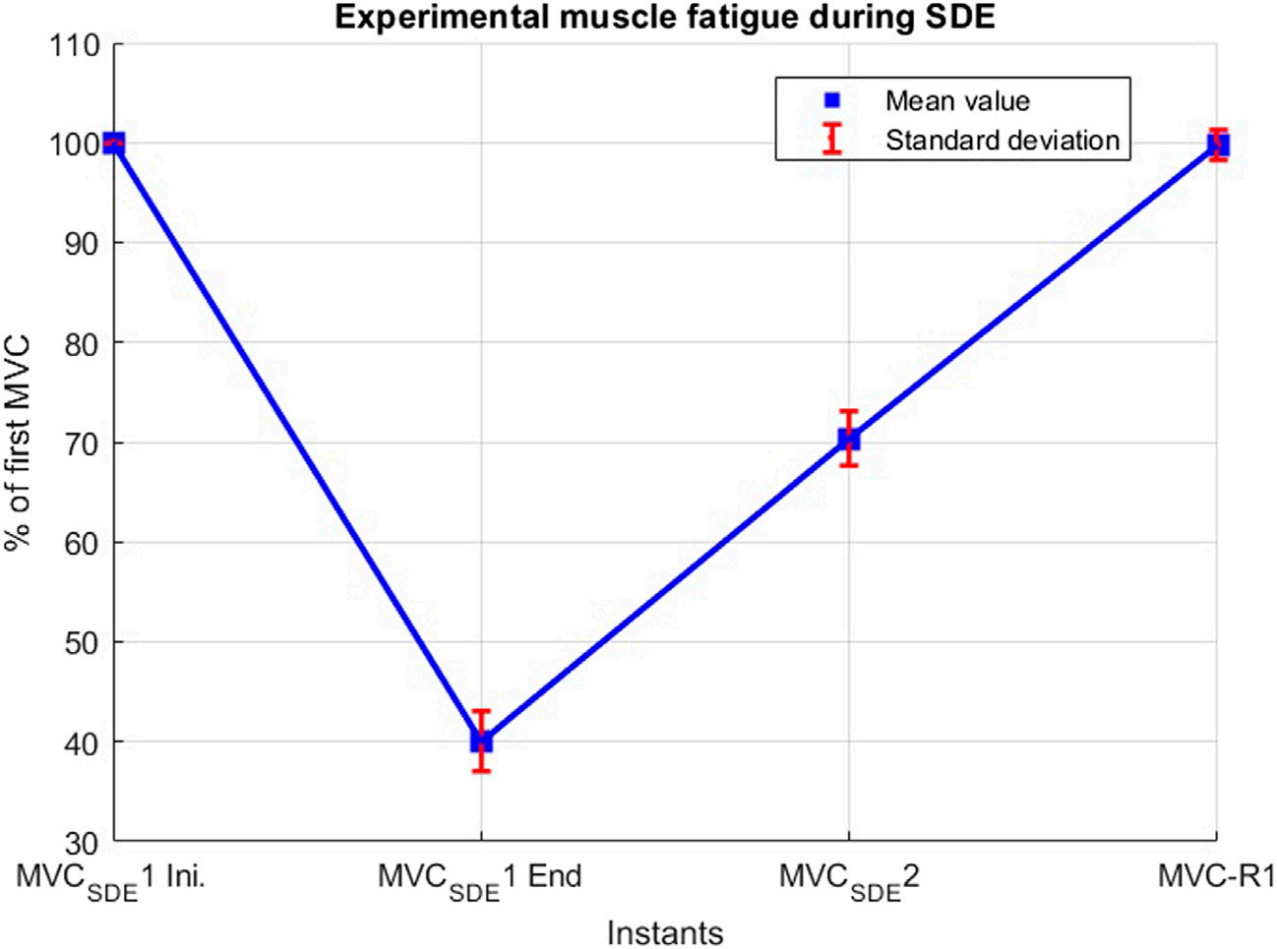
Experimental muscle fatigue during the short-duration exercise (SDE).

During the short-duration exercise, subjects exhibited an average force decay of 60% during MVC_SDE_1, and regained approximately 30% of this loss at MVC_SDE_2 after a 15-s recovery period (Figure 4). Subsequently, following a resting period of 10 min, the mean force measured at MVC-R1 reached 99.7%, with all subjects recovering between 93% and 104%.

**FIGURE 5:**
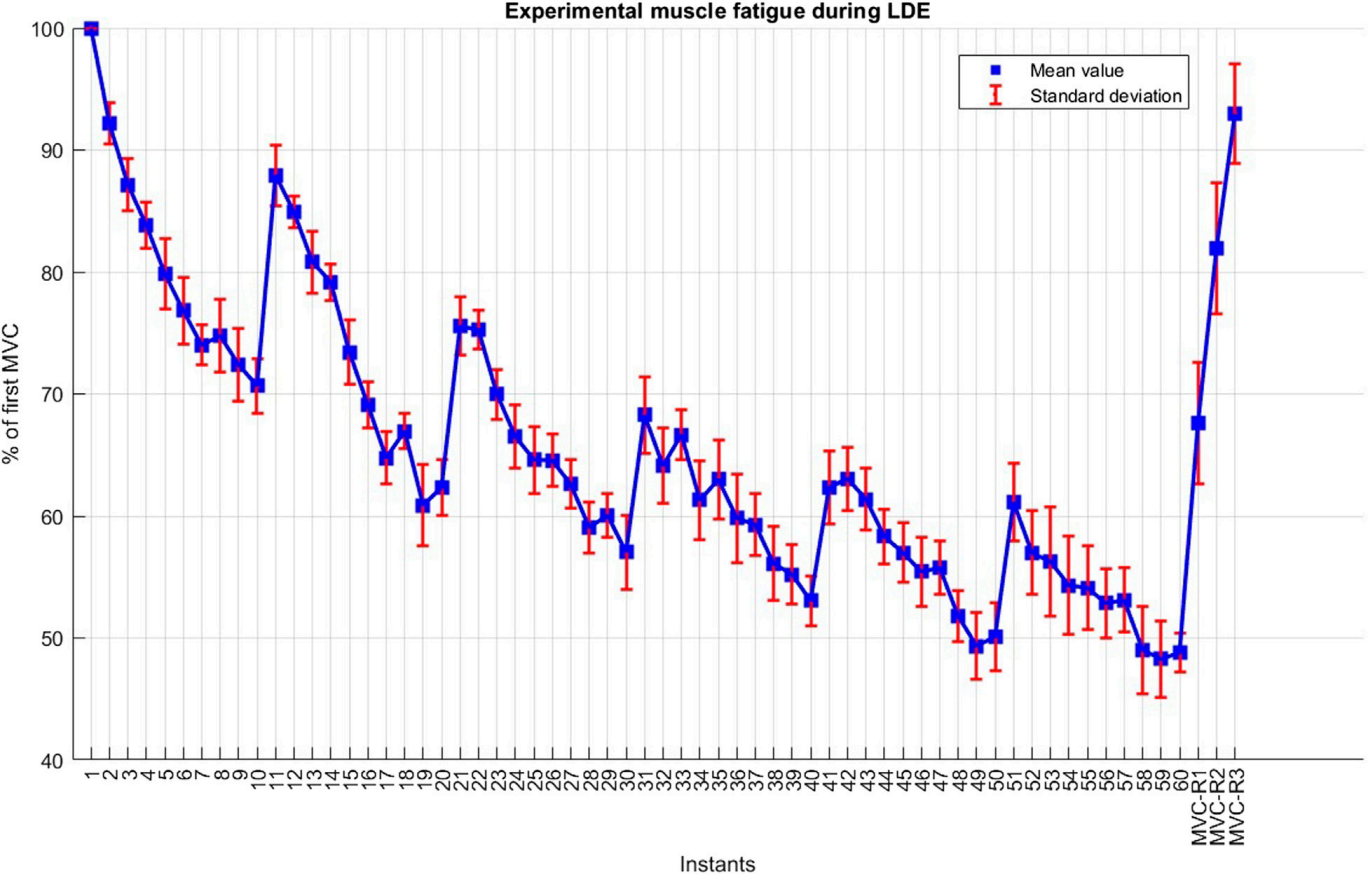
Experimental muscle fatigue during the long-duration exercise (LDE).

During the long-duration exercise, subjects demonstrated an average force decay of 51.2% following the sixty MVC_LDE_ (Figure 5). The 90-s resting periods introduced after the 10th, 20th, 30th, and 40th repetitions resulted in substantial recovery. The force decays during each set of ten repetitions were progressively less pronounced but consistently present. After resting periods of 10 min, 1 h, and 24 h, the mean forces measured at MVC-R1, MVC-R2, and MVC-R3 reached 67.6%, 81.9%, and 92.9%, respectively. Notably, only one subject achieved complete recovery (100%) after 24 h.

### 3.2 Model vs experimental comparisons

**FIGURE 6:**
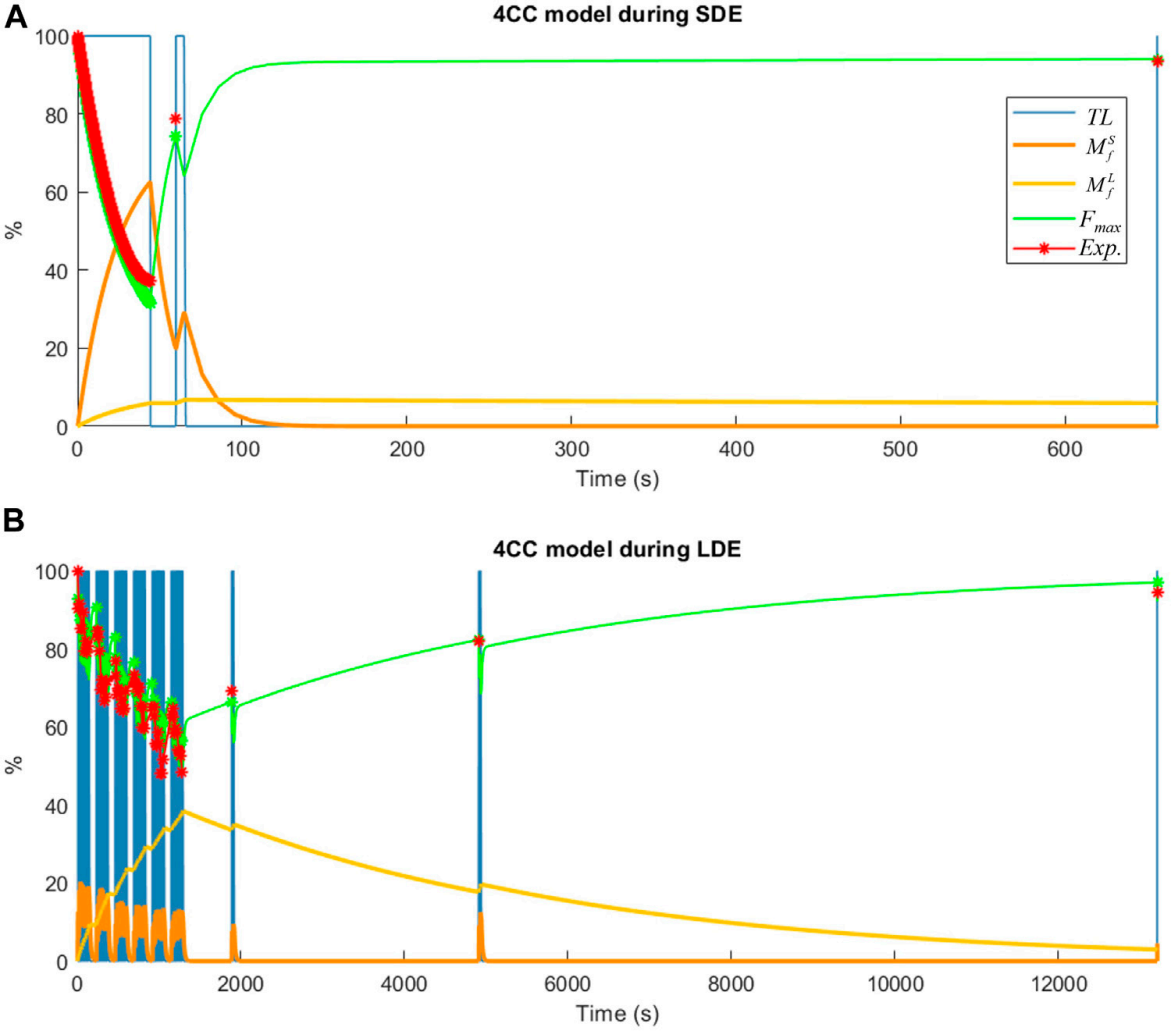
Muscle fatigue simulation of a subject using the 4CC model: **(A)** during short-duration exercise (SDE); **(B)** during long-duration exercise (LDE).

The simulated behavior of short-term fatigue ( $M_{f}^{S}$ ) and long-term fatigue ( $M_{f}^{L}$ ) in the new 4CC model is illustrated in Figure 6. It is evident that short-term fatigue (depicted in yellow) experienced rapid recovery, whereas long-term fatigue (depicted in orange) required a significantly longer rest period. The resulting available maximum force (*F*_max_) (Equation 6), depicted in green, showed a strong correlation with experimental measurements during maximum voluntary contractions (depicted in red).

**FIGURE 7:**
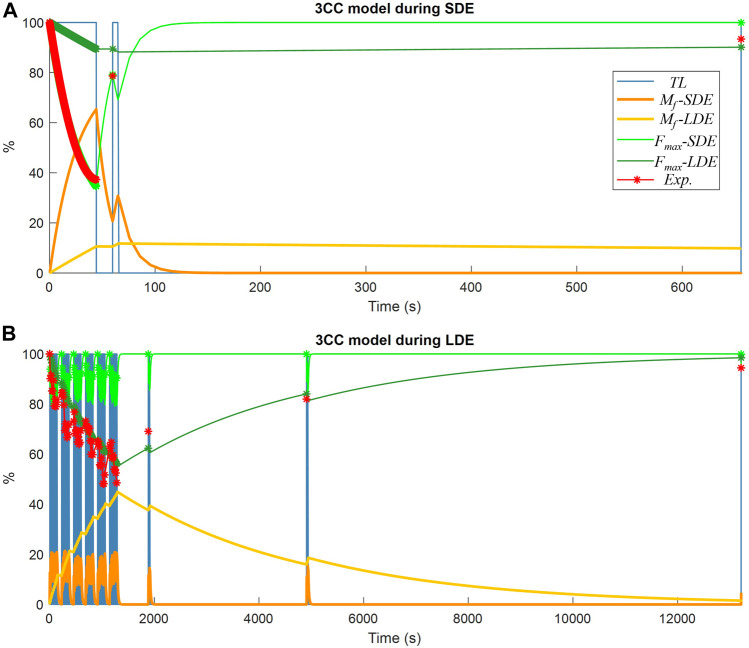
Muscle fatigue simulation of a subject using the 3CCr model: **(A)** during short-duration exercise (SDE); **(B)** during long-duration exercise (LDE).

The results obtained from the classical 3CCr approach, which served as benchmark in this study for validating the new model, are represented in Figure 7 using the two different calibration approaches, 3CC-SDE and 3CC-LDE, as explained previously. It is noticeable that the fatigue compartment *M*_*f*_ *-SDE* using short-term fatigue calibration exhibited rapid recovery, while the fatigue compartment *M*_*f*_ *-LDE* using long-term fatigue calibration required a significantly longer rest period. The resulting maximum forces available (*F*_*max*_ *-SDE* and *F*_*max*_ *-LDE* respectively) for each approach showed a good correlation during maximum voluntary contractions (depicted in red) with the experimental measurements used for the calibration of the corresponding approach, while significant differences were observed with the readings of the session not used for the calibration of the corresponding approach. Furthermore, when compared to the 4CC model, which accurately represented force variations throughout the series of the long-term exercise similar to the experimental measurements, the 3CC-LDE model exhibited only minor variations with an approximate mean value.

Mean RMSE between measured and simulated results across subjects for all the measurements (RMSE Tot.), and separately for the exercise phase (RMSE Ex.) and the recovery evaluations at MVC-R1 to MVC-R3 (RMSE Rec.), during the short-duration and long-duration exercise, are represented in Table 1. As noted earlier, the two 3CCr approaches, 3CC-SDE and 3CC-LDE, showed good correlations with the corresponding experimental measurements used for their respective calibrations, while significant errors were detected with the readings of the session not used for them. The 4CC model exhibited a reduced error rate for both sessions, with an overall RMSE of less than 6.0%. The largest error (7%) in the novel model occurred in estimating recovery during the short-duration exercise.

**TABLE 1:** Mean RMSE between measured and simulated results across subjects.

	Exercise					
	Short-duration exercise			Long-duration exercise		
	3CC-SDE	3CC-LDE	4CC	3CC-SDE	3CC-LDE	4CC
RMSE Tot. (%)	1.7	28.9	4.3	26.1	8.0	6.0
RMSE Ex. (%)	1.7	28.9	4.3	26.2	8.1	6.1
RMSE Rec. (%)	2.5	12.2	7.0	22.2	5.4	1.8

The mean values of the RMSE obtained during the two sessions with the three approaches are detailed in Table 2. The mean total error and recovery estimation error with the novel 4CC model were only 5.1% and 4.4%, respectively. In contrast, the classic 3CCr model displayed higher errors, with a total error of 13.9% for 3CC-SDE and 18.4% for 3CC-LDE, respectively. Consequently, the improvement offered by using the additional compartment was 8.8% for 3CC-SDE and 13.3% for 3CC-LDE. The paired sampled *t*-test revealed statistically significant differences (*p* < 2.2%) between the 4CC model and the previous ones.

**TABLE 2:** Comparison of the 3CC and 4CC models.

		Models		
		3CC-SDE	3CC-LDE	4CC
SDE-LDE Mean values	RMSE Tot. (%)	13.9	18.4	5.1
	RMSE Ex. (%)	14.0	18.5	5.2
	RMSE Rec. (%)	12.4	8.8	4.4
4CC differences	RMSE Tot. (%)	8.8	13.3	—
	RMSE Ex. (%)	8.8	13.3	—
	RMSE Rec. (%)	8.0	4.4	—
Paired sample *t*-test with 4CC	*p*-value Tot. (%)	0.01	0.11	—
	*p*-value Ex. (%)	0.01	0.11	—
	*p*-value Rec. (%)	2.14	1.34	—

## 4 Discussion

The aim of this investigation is to refine the existing 3CCr muscle fatigue model by incorporating an additional force decay component related to central fatigue (often referred to as “brain effort”) and a “long-term fatigue state” (fatigue from which the subject recovers only after an extended resting period). The authors introduced a novel four-compartment model that differentiates between the short-term fatigued state (linked to metabolic inhibition) and the long-term fatigued state (mimicking central fatigue and potential microtraumas).

Their recent experimental measurements during both short- and long-duration exercises confirmed the observations reported by Baker et al. (Baker et al., 1993). Despite similar levels of muscle fatigue, the time required for complete recovery depended on the duration of the preceding exercise. Various mechanisms contribute to fatigue, on one hand, short-duration exercise is primarily associated with metabolic inhibition. Changes in PCr (phosphocreatine) and Pi (inorganic phosphate) concentrations reflect the dynamic processes of ATP synthesis and breakdown during muscle contraction. As fatigue sets in, the balance between energy demand and regeneration becomes disrupted, leading to alterations in PCr and Pi levels. Monitoring these changes has provided insights into the metabolic state of muscles during physical activity and has helped understand the mechanisms underlying muscle fatigue (Levy et al., 1993; Baker et al., 1993). On the other hand, long-duration exercise involves an additional long-lasting nonmetabolic component that acts beyond the cell membrane, specifically at the level of excitation-contraction coupling (Baker et al., 1993). Jubeau et al. observed the development of delayed onset muscle soreness (DOMS) and increases in plasma creatine kinase (CK) following prolonged exercises, in the next 48 h for voluntary contraction, and in the next 96 h for electrically evoked contractions (Jubeau et al., 2012). The mean force measured at MVC-R1 after a 10-min rest reached 99.7% for the short-duration exercise, whereas the mean force measured at MVC-R3 after a 24-h rest reached 92.9% for the long-duration exercise. Jubeau et al. reported similar results in their long-duration experiments involving isometric contractions of the elbow flexors (Jubeau et al., 2012).

In this work, the authors introduced an innovative methodology to estimate subject-specific fatigue parameters for their model. The results obtained from the classical 3CCr approach served as a benchmark in this study for validating the novel model, using two different calibration approaches, 3CC-SDE and 3CC-LDE. Estimated maximum muscle forces were compared with strain gauge readings during MVCs, as it is the best way to assume that all muscles are fully activated during a maximum effort, thus avoiding the muscle force-sharing problem and the uncertainty about the level of effort made by the subject. The RMSE was evaluated during both short-duration and long-duration exercises. The resulting available maximum forces from the 3CC models showed good correlation with the experimental data of the session used for the calibration of each of them, while significant differences were observed with the readings of the other session. The novel 4CC model proposed by the authors demonstrated a strong correlation with the measurements of both sessions.

The mean RMSE between measured and simulated results across subjects validates the benefits of adding the fourth compartment, providing a mean total error and recovery estimation error of only 5.1% and 4.4%, respectively. The paired sampled *t*-test showed statistically significant differences between the 4CC model and the previous ones, with a confidence level exceeding 99.8% for the complete evaluation. The overall mean improvement was 8.8% with respect to 3CC-SDE and 13.3% with respect to 3CC-LDE. The validation procedure revealed the shortcomings of the classic 3CC in accurately matching target loads within a single muscle or at the joint level, highlighting its inadequacy in handling dynamic force profiles effectively. Nevertheless, it should be noted that the 3CC-SDE approach yielded better results than 4CC for the short-duration exercise simulation. The authors hypothesized that, during this brief session, long-term fatigue effects could be omitted; hence, the corresponding fourth compartment was not considered for calibration. It is noticeable that some long-term fatigue was generated during the SDE simulation, contributing to an increase in the total estimated fatigue. For a more accurate estimate, it is advisable to consider some long-term fatigue in the calibration model.

As a limitation of the study, the experiments focused on isometric contractions of the elbow flexors. The decision to limit the study to isometric motion was made to avoid potential errors introduced by moment arms variation if their calibration is not accurate (Michaud et al., 2023). The use of a single joint was intentional, as the authors plan to investigate in future work how central fatigue generated by a single joint can affect the fatigue of other joints. Similarly, they need to investigate how central fatigue can be incorporated while addressing the force-sharing problem between muscles (Michaud et al., 2021; Michaud et al., 2023). This will allow them to implement muscle fatigue behavior into their real-time human motion capture, reconstruction, and musculoskeletal analysis (Lugrís et al., 2023) in the future. Finally, given that muscle fatigue can vary across muscles and joints, the subject-specific model calibration proposed in this study must be applied to each individual joint, allowing for an accurate representation of the corresponding muscle fatigue behavior. Future studies may focus on determining mean model parameters using a larger sample.

## 5 Conclusion

The authors improved the existing 3CCr muscle fatigue model by incorporating an additional component of force decay related to central fatigue and a long-term fatigue state. The novel four-compartment model was set to differentiate between the short-term fatigued state (linked to metabolic inhibition) and the long-term fatigued state (mimicking central fatigue and potential microtraumas). Through recent experimental measurements during both short- and long-duration exercises, they validated their approach and also demonstrated the limitations of the classic 3CCr in handling any time-varying force profile.

## Data Availability

The raw data supporting the conclusion of this article will be made available by the authors, without undue reservation.
