# Correction: Four-compartment muscle fatigue model to predict metabolic inhibition and long-lasting nonmetabolic components

**DOI:** 10.3389/fphys.2025.1682045

**Published:** 2025-09-01

**Authors:** Florian Michaud, Santiago Beron, Urbano Lugrís, Javier Cuadrado

**Affiliations:** Laboratory of Mechanical Engineering, Centro de Investigación en Tecnologías Navales e Industriales (CITENI), Campus Industrial de Ferrol, Universidade da Coruña, Ferrol, Spain

**Keywords:** musculotendon dynamics, muscle force, muscle fatigue model, force prediction, musculotendon model, sport performance, ergonomics, mathematical models

In the published article, Equation 2, in Section 2 Material and methods, Subsection 2.2 Four-compartment muscle fatigue model, was erroneously given as $\frac{dMa}{dt}=C\left(t\right)-\left(R_{S}+R_{L}\right)\times M_{a}$ .

The correct equation is $\frac{dMa}{dt}=C\left(t\right)-\left(F_{S}+F_{L}\right)\times M_{a}$ .

The original article has been updated.

